# Earplug use during sleep and its association with cardiovascular disease – Results from a large sample of the general population

**DOI:** 10.1016/j.ijcha.2025.101642

**Published:** 2025-03-07

**Authors:** Omar Hahad, Volker H. Schmitt, Rieke Baumkötter, Matthias Michal, Julian Chalabi, Alexander K. Schuster, Emilio Gianicolo, Karl J. Lackner, Katharina Geschke, Julia Weinmann-Menke, Stavros Konstantinides, Andreas Daiber, Philipp S. Wild, Thomas Münzel

**Affiliations:** a Department of Cardiology – Cardiology I, University Medical Center of the Johannes Gutenberg-University Mainz, Mainz, Germany; b German Center for Cardiovascular Research (DZHK), partner site Rhine-Main, Mainz, Germany; c Preventive Cardiology and Preventive Medicine, Department of Cardiology, University Medical Center of the Johannes Gutenberg-University Mainz, Mainz, Germany; d Department of Psychosomatic Medicine and Psychotherapy, University Medical Center of the Johannes Gutenberg-University Mainz, Mainz, Germany; e Department of Ophthalmology, University Medical Center of the Johannes Gutenberg-University Mainz, Mainz, Germany; f Institute of Medical Biostatistics, Epidemiology & Informatics, University Medical Center of the Johannes Gutenberg-University Mainz, Mainz, Germany; g Institute of Clinical Chemistry and Laboratory Medicine, University Medical Center of the Johannes Gutenberg-University Mainz, Mainz, Germany; h Department of Psychiatry and Psychotherapy, University Medical Center of the Johannes Gutenberg-University Mainz, Mainz, Germany; i Department of Nephrology, I. Department of Medicine, University Medical Center of the Johannes Gutenberg University Mainz, Mainz, Germany; j Center for Thrombosis and Hemostasis, University Medical Center of the Johannes Gutenberg-University Mainz, Mainz, Germany; k Institute of Molecular Biology (IMB), Mainz, Germany

**Keywords:** Earplug use, Hearing protection, Noise annoyance, Cardiovascular disease, General population

## Abstract

**Introduction:**

Environmental factors contribute to cardiovascular disease (CVD) pathogenesis. Noise annoyance is linked to adverse cardiovascular outcomes, and earplug use may mitigate noise-related health effects. This study examines associations between earplug use, noise annoyance, and CVD in a general population sample.

**Methods:**

Cross-sectional data from 15,010 participants (35–74 years, 2007–2012) in the German Gutenberg Health Study were analyzed. Noise annoyance from various sources and earplug use during sleep were self-reported. Prevalent CVD (atrial fibrillation, coronary artery disease, myocardial infarction, stroke, chronic heart failure, peripheral artery disease, or venous thromboembolism) was determined through self-report or medical records. Logistic regression analyses with sequential adjustments evaluated associations.

**Results:**

Among 14,675 participants with earplug data, 713 (4.9%) reported use. Earplug users were more likely younger women with lower cardiovascular risk profiles but higher noise annoyance (90.3% vs. 78.8%). Logistic regression revealed consistent positive associations between earplug use and prevalent CVD across various noise sources, persisting after adjustments for covariates. For example, the odds ratio for earplug use and prevalent CVD, adjusted for neighborhood noise annoyance and other factors, was 1.91 (95% CI 1.39–2.59). No interaction between earplug use and noise annoyance was found.

**Conclusions:**

This study provides valuable insights into the relationships between earplug use, noise annoyance, and CVD. Findings highlight the need for targeted interventions addressing noise-related cardiovascular risks and emphasize the complex dynamics of these factors in cardiovascular health.

## 1 Introduction

Cardiovascular disease (CVD) remains a significant global health concern, with its multifaceted etiology continuing to be explored [1]. Environmental factors, such as noise exposure, have emerged as potential contributors to CVD development [2]. Noise annoyance, resulting from environmental noise exposure, has been linked to adverse cardiovascular outcomes [3], [4]. Concurrently, earplug use, a common method to mitigate noise exposure, has gained attention as a potential intervention to modify cardiovascular risk.

While previous studies have independently investigated the impact of noise annoyance on cardiovascular health [3], [4] and the efficacy of earplug use in noise reduction and prevention of hearing-related outcomes [5], [6], the interplay between these factors remains less explored. Understanding whether using earplugs can effectively mitigate the cardiovascular risks associated with noise annoyance represents a critical gap in the current literature. Indeed, there is evidence suggesting that the use of earplugs or hearing protection may exert an influence on outcomes implicated in mediating noise-induced CVD. In an early laboratory study from 1986, the influence of traffic noise on sleep in young male adults was examined [7]. The authors demonstrated that noise exposure led to lighter sleep and induced awakening effects. Importantly, the night with earplugs was largely undisturbed. Subsequent experimental studies have substantiated the favorable impact of nocturnal earplug use on both sleep quality and related factors [8], [9], [10], [11], [12], [13]. This is of particular importance, as nighttime noise is considered especially detrimental to cardiovascular health, exerting its impact by disturbing sleep [14]. In contrast, a study involving 1,587 healthy male blue-collar workers indicated that the use of hearing protection devices was associated with higher noise annoyance despite being associated with lower noise levels [15]. The authors concluded that in highly annoyed workers, hearing protection devices may be an additional source of stress and that future studies should focus on unannoyed workers who tend to use hearing protection devices less. However, in female workers, the catecholamine response to noise was reduced through earplugs, indicating the effective modulation of noise-induced stress pathways [16]. In a comprehensive study from Gopinath et al., the cross-sectional and longitudinal relationship between self-reported workplace noise exposure, use of hearing protection, and CVD in 2,932 workers was examined. While in individuals without use of hearing protection, workplace noise exposure was associated with 53 % and 75 % higher odds of prevalent CVD and angina, respectively, hearing protection use was associated with marginally reduced odds of having CVD and angina (odds ratio (OR) 0.49, 95 % confidence interval (CI) 0.24–1.01 and OR 0.38, 95 % CI 0.14–0.99, respectively) [17].

This study employs a comprehensive approach, integrating epidemiological data with subjective reports of earplug use during sleep and noise annoyance, to address the research gap by examining the association between earplug use, noise annoyance, and CVD in a large general population sample.

## 2 Methods

*Study design and sample*.

The present study analyzed data from the Gutenberg Health Study (GHS), the details of which, including study design and procedures, have been previously documented [18]. In brief, the GHS is a population-based, prospective, single-center cohort study conducted at the University Medical Center Mainz in Midwestern Germany. The main objective is to identify risk factors and causes of major common diseases and contribute to prevention. Participants were randomly selected from registration offices and invited by letter. Of 210,867 eligible residents of Mainz and Mainz-Bingen, 35,008 were randomly selected and stratified by sex, age, and residence. Exclusion criteria included insufficient German proficiency or inability to participate. The final enrollment was 15,010 (aged 35 to 74), with a response rate of 60.3 %. The baseline assessment took place from April 2007 to March 2012 and included a 5-hour interdisciplinary examination comprising anthropometric, cardiovascular, psychological, and ophthalmological tests, as well as computer-assisted interviews on psychosocial, environmental, and lifestyle factors. Ethical approval for all procedures conducted within the GHS was granted by the ethics committee of the Statutory Physician Board of the State Rhineland-Palatinate (reference number 2018–13720), and adherence to ethical principles outlined in the Declaration of Helsinki for medical research involving human subjects was strictly maintained. Written informed consent was obtained from all participants before their inclusion.

*Noise annoyance and use of earplugs*.

Self-reported noise annoyance was assessed using a standardized and validated approach, as described elsewhere [3]. Respondents utilized a 5-point Likert scale, encompassing responses ranging from “not at all” to “extremely,” to rate the extent of annoyance experienced over the preceding years in relation to various noise sources encountered during the day or sleep. These sources included road traffic, aircraft, railway, industrial, and neighborhood noise. The composite measure of total noise annoyance was determined by identifying the highest reported annoyance rating (>0), irrespective of the specific noise source and whether the annoyance occurred during the day or sleep. Similarly, source-specific total noise annoyance was defined as the maximum rating associated with a specific noise source, regardless of its impact on daytime or sleep-related experiences.

Earplug use was evaluated through a standardized questionnaire employing a binary response format, wherein participants were asked, “do you sleep with earplugs or similar?” with response options limited to “yes” or “no”.

*Prevalent cardiovascular disease*.

The determination of prevalent CVD was conducted through self-report, examination of medical records involving physician diagnoses, or diagnoses established during study visits. Prevalent CVD was defined by the presence of any of the following medical conditions: atrial fibrillation, coronary artery disease, myocardial infarction, stroke, chronic heart failure, peripheral artery disease, and venous thromboembolism.

*Covariates*.

Data about sociodemographic variables, traditional cardiovascular risk factors, and drug intake were extracted from the extensive 5-hour baseline examination to implement a thorough statistical adjustment strategy. Precise definitions for the covariates used in this study are available in **Supplementary Table S1**.

### 2.1 Statistical analysis

Characteristics of the study sample are shown stratified by earplug use as mean and standard deviation for continuous variables, or if skewness >1 by median (Q1, Q3). Binary variables are described as relative and absolute frequencies. Logistic regression analysis with corresponding OR and 95 % CI were used to estimate the relationship between earplug use and prevalent CVD (composite of atrial fibrillation, coronary artery disease, myocardial infarction, stroke, chronic heart failure, peripheral artery disease, and venous thromboembolism). Distinct analyses were conducted to account for adjustments related to each source of noise annoyance, alongside an additional interaction analysis examining source-specific noise annoyance and earplug use. Furthermore, we conducted a stratification analysis in individuals with and without noise annoyance. Statistical analysis included sequential adjustment. Model 1 was adjusted for sex (binary) and age (continuous). Model 2 was additionally adjusted for socioeconomic status (continuous), diabetes mellitus (binary), arterial hypertension (binary), current smoking (binary), obesity (binary), dyslipidemia (binary), family history of myocardial infarction or stroke (binary), and noise annoyance (continuous). Model 3 was additionally adjusted for medication use (diabetic drugs, antithrombotic agents, antihypertensives, diuretics, beta-blockers, calcium channel blockers, agents acting on the renin-angiotensin-aldosterone system, and lipid modifying agents, all binary) and the interaction term of use of earplugs and noise annoyance (continuous). The statistical data analyses were performed using the software R version 4.2.1 (https://www.r-project.org/).

## 3 Results

*Study sample characteristics*.

After excluding participants without information on earplug use, a total of *N* = 14,675 participants were included in the present analysis. Among these, 713 (4.9 %) reported to use earplugs during sleep (Table 1). Earplug users were characterized by a higher proportion of women (63.8 %) and a younger age profile (mean age of 52.5 years) compared to non-users (48.7 % women, mean age of 55.0 years). Regarding cardiovascular risk factors, non-earplug users exhibited higher percentages of current smoking (19.6 %), diabetes mellitus (9.3 %), arterial hypertension (49.8 %), obesity (25.4 %), and dyslipidemia (34.6 %). Further investigation into cardiovascular medication usage revealed that earplug users reported lower proportions of medication across several categories. However, regarding noise annoyance, earplug users reported higher annoyance (>0) for various sources (road traffic, aircraft, railway, industrial, and neighborhood) both during the day and while sleeping. The overall total noise annoyance was higher among earplug users (90.3 %) compared to non-users (78.8 %). The prevalence of CVD was higher among earplug users (32.0 %) compared to non-earplug users (26.6 %),

**Table 1:** Characteristics of the study sample stratified by earplug use (*N* = 14,675).

	No earplug use (n = 13,962)	Earplug use (n = 713)	
Women – % (no.)	48.7 (6,796)	63.8 (455)	
Age – years	55.0 (11.1)	52.5 (10.8)	
Socioeconomic status (SES) – score	12.87 (4.48)	13.71 (4.28)	
Working night shift – % (no.)	12.9 (1,023)	18.3 (83)	
Time living at current residence – years	16.00 (8.00/30.00)	12.00 (6.00/25.00)	
*Traditional cardiovascular risk factors*			
Current smoking – % (no.)	19.6 (2,738)	16.3 (116)	
Diabetes mellitus – % (no.)	9.3 (1,295)	4.5 (32)	
Arterial hypertension – % (no.)	49.8 (6,956)	41.2 (294)	
Obesity – % (no.)	25.4 (3,547)	17.1 (122)	
Dyslipidemia – % (no.)	34.6 (4,822)	29.1 (207)	
Family history of myocardial infarction or stroke – % (no.)	21.9 (3,053)	23.1 (165)	
*Cardiovascular disease*			
Any cardiovascular disease – % (no.)	26.6 (3,695)	32.0 (228)	
*Cardiovascular medication*			
Diabetic drugs (A10) – % (no.)	6.1 (847)	3.0 (21)	
Antithrombotic agents (B01) – % (no.)	12.2 (1,681)	9.3 (66)	
Antihypertensives (C02) – % (no.)	1.0 (143)	0.7 (5)	
Diuretics (C03) – % (no.)	5.3 (743)	2.3 (16)	
Beta-blockers (C07) – % (no.)	17.0 (2,352)	12.3 (87)	
Calcium channel blocker (C08) – % (no.)	7.3 (1,012)	5.4 (38)	
Agents acting on the renin-angiotensin-aldosterone system (C09) – % (no.)	23.7 (3,278)	18.5 (131)	
Lipid modifying agents (C10) – % (no.)	13.4 (1,848)	9.6 (68)	
*Noise annoyance*			
Road traffic noise annoyance (>0, day) – % (no.)	40.8 (5,679)	50.0 (355)	
Aircraft noise annoyance (>0, day) – % (no.)	57.9 (8,060)	67.9 (482)	
Railway noise annoyance (>0, day) – % (no.)	14.4 (2,004)	16.8 (119)	
Industrial noise annoyance (>0, day) – % (no.)	13.2 (1,842)	17.6 (125)	
Neighborhood noise annoyance (>0, day) – % (no.)	35.3 (4,915)	48.9 (347)	
Road traffic noise annoyance (>0, sleep) – % (no.)	15.7 (2,182)	29.4 (208)	
Aircraft noise annoyance (>0, sleep) – % (no.)	30.7 (4,267)	46.6 (330)	
Railway noise annoyance (>0, sleep) – % (no.)	7.9 (1,095)	11.7 (83)	
Industrial noise annoyance (>0, sleep) – % (no.)	2.5 (341)	5.7 (40)	
Neighborhood noise annoyance (>0, sleep) – % (no.)	15.3 (2,124)	35.0 (248)	
Total noise annoyance (>0) – % (no.)	78.8 (10,972)	90.3 (641)	

Continuous variables are shown as mean and standard deviation or if skewness > 1 by median (Q1, Q3). Binary variables are described as relative and absolute frequencies.

Socioeconomic status score ranges from 3 to 21 with higher values indicating higher status.

Medication is labelled with the anatomical therapeutic chemical-code.

The presence of cardiovascular disease was defined by any of the following: atrial fibrillation, coronary artery disease, myocardial infarction, stroke, chronic heart failure, peripheral artery disease, or venous thromboembolism.

*Association between use of earplugs and prevalent cardiovascular disease*.

Table 2 shows the results of the logistic regression analysis concerning the association between earplug use and prevalent CVD. In general, consistent positive associations between earplug use and prevalent CVD in the fully adjusted models, including adjustment for (source-specific) total noise annoyance, were observed. For instance, after adjustment for total aircraft noise annoyance, individuals who reported earplug use experienced 44 % higher odds of prevalent CVD compared to those who did not use earplugs (OR 1.44, 95 % CI 1.01–2.03). Similar substantial increases in prevalence persisted following adjustment for annoyance due to total road traffic (OR 1.64, 95 % CI 1.21–2.20), railway (OR 1.68, 95 % CI: 1.31–2.13), industrial (OR 1.70, 95 % CI 1.33–2.18), and neighborhood noise (OR 1.91, 95 % CI 1.39–2.59).

**Table 2:** Cross-sectional association analysis between use of earplugs and prevalent cardiovascular disease (data from the Gutenberg Health Study 2007–2012).

	*N*	Model 1		Model 2		Model 3		Interaction
		OR [95 % CI]		OR [95 % CI]		OR [95 % CI]		*P* value
(A)	8,203	1.41 [1.19; 1.67]		1.67 [1.34; 2.07]		1.64 [1.21; 2.20]		0.92
(B)	8,206			1.67 [1.34; 2.07]		1.44 [1.01; 2.03]		0.31
(C)	8,197			1.68 [1.35; 2.08]		1.68 [1.31; 2.13]		0.89
(D)	8,198			1.65 [1.33; 2.05]		1.70 [1.33; 2.18]		0.45
(E)	8,199			1.57 [1.26; 1.95]		1.91 [1.39; 2.59]		0.069
(F)	8,207			1.57 [1.26; 1.95]		1.50 [0.91; 2.42]		0.83

Odds ratios (OR) and 95% confidence intervals (CI) are derived from a logistic regression model modeling for prevalent cardiovascular disease (CVD, composite variable comprising atrial fibrillation, coronary artery disease, myocardial infarction, stroke, chronic heart failure, peripheral artery disease, and venous thromboembolism). *P* value for interaction of noise annoyance and use of earplugs.

*N* denotes model 3.

Model 1 was adjusted for sex and age.

Model 2 was additionally adjusted for socioeconomic status, night shift work, years lived in residence, and noise annoyance ((A): with adjustment for total road traffic noise annoyance, (B) with adjustment for total aircraft noise annoyance, (C) with adjustment for total railway noise annoyance, (D) with adjustment for total industrial noise annoyance, (E) with adjustment for total neighborhood noise annoyance, (F) with adjustment for total noise annoyance).

Model 3 was additionally adjusted for diabetes mellitus, arterial hypertension, smoking, obesity, dyslipidemia, family history of myocardial infarction or stroke, medication use (diabetic drugs, antithrombotic agents, antihypertensives, diuretics, beta-blockers, calcium channel blocker, agents acting on the renin-angiotensin-aldosterone system, and lipid modifying agents), and the interaction term of earplug use and noise annoyance.

The results did not reveal evidence supporting the presence of an interaction effect between earplug use and (source-specific) total noise annoyance in influencing the prevalence of CVD.

*Stratification analysis in individuals with and without noise annoyance*.

Table 3, Table 4 display the results of the stratification analysis in individuals with (>0) and without (source-specific, =0) total noise annoyance. Again, consistent positive associations between earplug use and prevalence of CVD in the fully adjusted models in individuals with and without (source-specific) total noise annoyance were observed. For instance, in individuals with road traffic noise annoyance, earplug users experienced 63 % higher odds of prevalent CVD (OR 1.63, 95 % CI 1.19–2.20), a trend that intensified to a 74 % increase in the absence of road traffic noise annoyance (OR 1.74, 95 % CI 1.24–2.40). In the setting of neighborhood noise annoyance, earplug users exhibited a 43 % increase in odds (OR 1.43, 95 % CI 1.06–1.91) when annoyance was present, and a 99 % increase was observed in its absence (OR 1.99, 95 % CI 1.39–2.81).

**Table 3:** Cross-sectional association analysis between use of earplugs and prevalent cardiovascular disease in individuals with noise annoyance (data from the Gutenberg Health Study 2007–2012).

	*N*	Model 1		Model 2		Model 3	
		OR [95 % CI]		OR [95 % CI]		OR [95 % CI]	
Total road traffic noise annoyance (>0)	3,618	1.34 [1.06; 1.68]		1.63 [1.21; 2.19]		1.63 [1.19; 2.20]	
Total aircraft noise annoyance (>0)	4,958	1.37 [1.11; 1.67]		1.83 [1.41; 2.36]		1.87 [1.44; 2.43]	
Total railway noise annoyance (>0)	1,372	1.21 [0.81; 1.77]		1.57 [0.96; 2.52]		1.39 [0.82; 2.29]	
Total industrial noise annoyance (>0)	1,352	1.00 [0.65; 1.50]		1.40 [0.85; 2.22]		1.39 [0.83; 2.25]	
Total neighborhood noise annoyance (>0)	3,713	1.29 [1.03; 1.62]		1.43 [1.07; 1.89]		1.43 [1.06; 1.91]	
Total noise annoyance (>0)	6,636	1.39 [1.16; 1.66]		1.67 [1.32; 2.09]		1.65 [1.30; 2.08]	

Odds ratios (OR) and 95% confidence intervals (CI) are derived from a logistic regression model modeling for prevalent cardiovascular disease (CVD, composite variable comprising atrial fibrillation, coronary artery disease, myocardial infarction, stroke, chronic heart failure, peripheral artery disease, and venous thromboembolism).

*N* denotes model 3.

Model 1 was adjusted for sex and age.

Model 2 was additionally adjusted for socioeconomic status, night shift work, and years lived in residence.

Model 3 was additionally adjusted for diabetes mellitus, arterial hypertension, smoking, obesity, dyslipidemia, family history of myocardial infarction or stroke, and medication use (diabetic drugs, antithrombotic agents, antihypertensives, diuretics, beta-blockers, calcium channel blocker, agents acting on the renin-angiotensin-aldosterone system, and lipid modifying agents).

**Table 4:** Cross-sectional association analysis between use of earplugs and prevalent cardiovascular disease in individuals without noise annoyance (data from the Gutenberg Health Study 2007–2012).

	*N*	Model 1		Model 2		Model 3	
		OR [95 % CI]		OR [95 % CI]		OR [95 % CI]	
Total road traffic noise annoyance (=0)	4,588	1.48 [1.15; 1.88]		1.76 [1.27; 2.41]		1.74 [1.24; 2.40]	
Total aircraft noise annoyance (=0)	3,245	1.54 [1.14; 2.07]		1.39 [0.91; 2.08]		1.24 [0.78; 1.91]	
Total railway noise annoyance (=0)	6,825	1.45 [1.20; 1.74]		1.72 [1.34; 2.18]		1.75 [1.35; 2.24]	
Total industrial noise annoyance (=0)	6,846	1.50 [1.25; 1.80]		1.77 [1.38; 2.25]		1.75 [1.35; 2.24]	
Total neighborhood noise annoyance (=0)	4,486	1.44 [1.12; 1.85]		2.01 [1.42; 2.81]		1.99 [1.39; 2.91]	
Total noise annoyance (=0)	1,571	1.32 [0.75; 2.24]		1.65 [0.77; 3.30]		1.77 [0.80; 3.65]	

Odds ratios (OR) and 95% confidence intervals (CI) are derived from a logistic regression model modeling for prevalent cardiovascular disease (CVD, composite variable comprising atrial fibrillation, coronary artery disease, myocardial infarction, stroke, chronic heart failure, peripheral artery disease, and venous thromboembolism).

*N* denotes model 3.

Model 1 was adjusted for sex and age.

Model 2 was additionally adjusted for socioeconomic status, night shift work, and years lived in residence.

Model 3 was additionally adjusted for diabetes mellitus, arterial hypertension, smoking, obesity, dyslipidemia, family history of myocardial infarction or stroke, and medication use (diabetic drugs, antithrombotic agents, antihypertensives, diuretics, beta-blockers, calcium channel blocker, agents acting on the renin-angiotensin-aldosterone system, and lipid modifying agents).

## 4 Discussion

The present study, conducted within the context of a large cohort of the general population and involving 14,675 participants, sought to examine the relationships between earplug use, noise annoyance, and prevalent CVD. Notably, earplug users constituted a distinct subgroup, comprising 4.9 % of the study sample. Non-earplug users displayed higher percentages of traditional risk factors such as current smoking, diabetes mellitus, arterial hypertension, obesity, and dyslipidemia. Earplug users reported lower rates of medication use across various categories. Earplug users reported higher annoyance levels for various noise sources during both daytime and sleep. This finding resulted in higher total noise annoyance among earplug users compared to non-users. Present findings indicated consistent positive associations between earplug use and prevalent CVD after adjustment for various sources of noise annoyance and in stratification analyses. The study did not find evidence supporting an interaction effect between earplug use and noise annoyance on prevalent CVD.

The relationship between noise exposure and annoyance, earplug use, and the risk of CVD unfolds through a complex interplay of physiological and psychological factors. Earplug use, commonly employed as a protective measure against environmental noise, aims to reduce auditory stimulation during sleep or in noisy settings [19]. Chronic exposure to elevated noise levels, a well-established cardiovascular risk factor, can contribute to physiological stress responses, leading to CVD [20]. A key question here is whether earplug use effectively modifies noise annoyance or merely serves as a marker for individuals who experience higher levels of noise annoyance. Understanding this distinction is crucial, as it may clarify whether earplug use represents a proactive coping mechanism against environmental noise or reflects heightened sensitivity and vulnerability to noise exposure. This dynamic could be influenced by psychological factors, including heightened sensitivity to noise or a sense of ineffectiveness in completely blocking out environmental sounds. Noise sensitivity is acknowledged as a general indicator of environmental sensitivity, which may influence health outcomes independent of actual noise exposure levels. Studies have shown that higher noise sensitivity is associated with increased blood pressure, cardiovascular problems, and greater use of medications [21]. Interestingly, Babisch et al. examined exposure modifiers in the HYENA study, which investigated the effects of aircraft and road traffic noise on hypertension [22]. The study found that the use of noise-reducing remedies, including earplugs, was more an indicator of perceived noise disturbance rather than an effective modifier that reduced noise annoyance or hypertension risk​. In line with our results, this may suggest that individuals who use earplugs may already perceive themselves as more sensitive to noise, rather than earplugs serving as an objective protective measure against noise-related health effects. In contrast, Olbrich et al. found in a prospective patient cohort study that patients with noise-proof windows had a significantly weaker association between aircraft noise exposure and recurrent CVD events, while those without insulation showed stronger associations [23]. However, this may also relate to the effectiveness of different noise mitigation strategies, where noise-proof windows reduce noise exposure consistently throughout the day and night, whereas using earplugs during sleep may not fully eliminate sleep disturbances and annoyance, as their effectiveness depends also on individual adherence and proper use. This is of special importance as noise annoyance during the day may also lead to increased physiological and psychological stress, which persists into the night, potentially affecting sleep quality, autonomic regulation, and cardiovascular health. Previous research has shown that individual daytime noise exposure was associated with immediate changes in heart rate variability, suggesting a possible mechanism linking noise to cardiovascular risk [24]. Moreover, in our analysis, individuals who use earplugs during sleep also reported higher noise annoyance during the day. This further suggests that earplug use may not be merely a reaction to noise annoyance during sleep but rather a marker of an overall heightened noise burden or sensitivity.

Our results may further find some explanation in previous evidence examining the relationship between personality traits and pain perception. Pud et al. revealed that individuals with high harm avoidance demonstrated heightened sensitivity to pain [25]. This heightened sensitivity may extend to other sensory experiences, including auditory processing. Therefore, individuals with high harm avoidance may be more susceptible to experiencing increased annoyance in response to noise, which aligns with the reported higher noise annoyance among earplug users. Additionally, higher harm avoidance was associated with an elevated stress response, contributing to increased pain sensitivity and potentially heightened annoyance in response to noise. The relationship between earplug use, noise annoyance, and cardiovascular risk underscores the importance of considering not only the physiological impact of noise but also the psychological responses that may contribute to adverse cardiovascular health effects. Noise annoyance, as a subjective response to environmental noise, is influenced by psychological and individual factors [26]. Persistent noise annoyance has been linked to stress-related disorders such as depression and anxiety [27], further emphasizing its potential role in cardiovascular outcomes. The psychological stress induced by chronic noise annoyance may activate pathways detrimental to cardiovascular health, contributing to the development or exacerbation of cardiovascular conditions [4]. While earplug use may provide a degree of protection against the direct physiological effects of noise exposure [5], [6], its impact on psychological factors, such as annoyance, requires careful consideration highlighting the need for a comprehensive understanding of both the physiological and psychological dimensions involved. It may be plausible that individuals experiencing noise annoyance may use earplugs regardless of the actual noise levels they encounter. This suggests that this subgroup, characterized by a heightened sensitivity to noise, could represent a vulnerable population more predisposed to CVD. In line with this, Laszlo et al. emphasized that noise annoyance is not solely determined by noise exposure but is also shaped by non-acoustic factors such as noise sensitivity, expectations, and trust in authorities [28]. They further highlight that noise reduction interventions, such as window insulation and noise barriers, do not necessarily lead to a proportional reduction in noise annoyance or improved well-being, stressing the complex effects on annoyance and health.

Despite the plausible hypothesis that hearing protection devices, such as earplugs, may mitigate noise-induced cardiovascular risks [16], [17], [7], [8], [9], [10], [11], [12], [13], our study results indicate a positive relationship between earplug use and prevalent CVD. Recent studies found that hearing protection devices, including earplugs, effectively reduce noise exposure, with a *meta*-analysis showing a significant sound attenuation effect (SMD 1.080, 95 % CI 0.167–1.993) [29]. In a randomized clinical trial, Ramakers et al. found that earplugs with an 18 dB noise reduction rate significantly reduced the risk of temporary threshold shifts after loud music exposure (8 % in the earplug group vs. 42 % in the unprotected group) and decreased newly induced tinnitus (12 % vs. 40 %) [5]. Interestingly, in an analysis of 1,390 occupational noise-exposed workers and 1,399 frequency matched non-noise-exposed individuals, there was no difference in risk of prevalent hypertension and blood pressure between earplug users and non-users [30]. We consistently observed an increased prevalence of CVD associated with earplug use, irrespective of noise annoyance status and after adjusting for various sources of noise annoyance. Our findings are more in line with previous research indicating that the use of hearing protection devices, including earplugs, is associated with heightened noise annoyance [15]. It is crucial to emphasize that direct comparisons with previous studies may be challenging due to the distinct nature of our analysis based on epidemiological data from the general population, whereas previous studies predominantly focused on specific samples within occupational or interventional design contexts.

To sum up the main limitations of our study: the cross-sectional and observational design of our study restricts our ability to establish causal relationships and discern the direction of causality. It is challenging to definitively determine whether individuals began using earplugs before or after developing CVD. Thus, the possibility of reverse causality emerges, suggesting that individuals experiencing the onset of CVD symptoms might initiate earplug use as a preventive measure to impede the progression of the disease. Additionally, the absence of specific data on noise exposure levels hinders our ability to precisely link noise annoyance with actual noise intensity. We have also no data on noise sensitivity, which may play an important role in the relationship between noise exposure and CVD [21]. Furthermore, no data were available on stress-related factors or general measures of psychological distress, which may have interfered with the results. The reliance on self-reported earplug use introduces the possibility of reporting bias, as the accuracy of such subjective measures is limited. Moreover, no information regarding the adherence or consistency in wearing earplugs among participants was available. Also, the study lacks specific data on the quality and type of earplugs used, including whether they were custom-made or standard [19]. These factors could introduce variability in the effectiveness of earplugs in mitigating noise exposure, and the absence of this detailed information may influence the interpretation of our results. Our study, focusing on the general population, may lack direct applicability to specific occupational or interventional contexts. Generalizability to samples studied in more specialized settings should be approached cautiously. Despite comprehensive statistical adjustments, the presence of unmeasured confounders cannot be ruled out, potentially influencing the observed associations.

Our study possesses several notable strengths. It leverages a comprehensive epidemiological approach using a large cohort representative of the general population. Thorough statistical adjustments were implemented for sociodemographic variables, cardiovascular risk factors, and medication use, enhancing the reliability of our analyses. We conducted an in-depth examination of noise annoyance patterns related to various sources. Our study reveals consistent associations between earplug use and prevalent CVD across various subgroups, demonstrating the robustness of our findings. Importantly, our study contributes to the identification of critical knowledge gaps in the existing literature.

In conclusion, this study provides insights into the relationship between earplug use, noise annoyance, and prevalent CVD, suggesting consistent positive associations between earplug use and prevalent CVD, even after adjusting for noise annoyance. The present results underscore the complexity of these factors, emphasizing the need for further exploration in longitudinal studies that consider the temporal dynamics of events and conditions. Future studies should consider the need for a comprehensive understanding of both the physiological and psychological dimensions involved in mitigating noise-related cardiovascular risk.

## CRediT authorship contribution statement

**Omar Hahad:** Writing – review & editing, Writing – original draft, Validation, Supervision, Project administration, Methodology, Investigation, Conceptualization. **Volker H. Schmitt:** Writing – review & editing, Writing – original draft, Validation, Supervision, Project administration, Investigation, Conceptualization. **Rieke Baumkötter:** Writing – review & editing, Writing – original draft. **Matthias Michal:** Writing – review & editing, Writing – original draft. **Julian Chalabi:** Writing – review & editing, Writing – original draft, Formal analysis. **Alexander K. Schuster:** Writing – review & editing, Writing – original draft. **Emilio Gianicolo:** Writing – review & editing, Writing – original draft. **Karl J. Lackner:** Writing – review & editing, Writing – original draft. **Katharina Geschke:** Writing – review & editing, Writing – original draft. **Julia Weinmann-Menke:** Writing – review & editing, Writing – original draft. **Stavros Konstantinides:** Writing – review & editing, Writing – original draft. **Andreas Daiber:** Writing – review & editing, Writing – original draft. **Philipp S. Wild:** Writing – review & editing, Writing – original draft. **Thomas Münzel:** Writing – review & editing, Writing – original draft.

## Declaration of competing interest

The authors declare that they have no known competing financial interests or personal relationships that could have appeared to influence the work reported in this paper.
