# Supplementary material for: Earplug use during sleep and its association with cardiovascular disease – Results from a large sample of the general population
Source: Int J Cardiol Heart Vasc. 2025 Mar 7;57:101642. doi: 10.1016/j.ijcha.2025.101642 (PMC11930695; doi:10.1016/j.ijcha.2025.101642)
Supplement: Supplementary Data 1 [file mmc1.docx]

**Earplug use during sleep and its association with cardiovascular disease – results from a large sample of the general population**

**Online Supplement**

Omar Hahad^1,2*^, Volker H. Schmitt^1,2*^, Rieke Baumkötter^2,3^, Matthias Michal^4^, Julian Chalabi^3^, Alexander K. Schuster^5^, Emilio Gianicolo^6^, Karl J. Lackner^7^, Katharina Geschke^8^, Julia Weinmann-Menke^9^, Stavros Konstantinides^10^, Andreas Daiber^1,2^, Philipp S. Wild^3,10,2,11^, Thomas Münzel^1,2^

^1^ Department of Cardiology – Cardiology I, University Medical Center of the Johannes Gutenberg-University Mainz, Mainz, Germany

^2^ German Center for Cardiovascular Research (DZHK), partner site Rhine-Main, Mainz, Germany

^3^ Preventive Cardiology and Preventive Medicine, Department of Cardiology, University Medical Center of the Johannes Gutenberg-University Mainz, Mainz, Germany

^4^ Department of Psychosomatic Medicine and Psychotherapy, University Medical Center of the Johannes Gutenberg-University Mainz, Mainz, Germany

^5^ Department of Ophthalmology, University Medical Center of the Johannes Gutenberg-University Mainz, Mainz, Germany

^6^ Institute of Medical Biostatistics, Epidemiology & Informatics, University Medical Center of the Johannes Gutenberg-University Mainz, Mainz, Germany

^7^ Institute of Clinical Chemistry and Laboratory Medicine, University Medical Center of the Johannes Gutenberg-University Mainz, Mainz, Germany

^8^ Department of Psychiatry and Psychotherapy, University Medical Center of the Johannes Gutenberg-University Mainz, Mainz, Germany

^9^ Department of Nephrology, I. Department of Medicine, University Medical Center of the Johannes Gutenberg University Mainz, Mainz, Germany

^10^ Center for Thrombosis and Hemostasis, University Medical Center of the Johannes Gutenberg-University Mainz, Mainz, Germany

^11^ Institute of Molecular Biology (IMB), Mainz, Germany

*Shared first authors.

**Address for correspondence:**

Dr. Omar Hahad

Department of Cardiology, Cardiology I

University Medical Center of the Johannes Gutenberg University Mainz

Langenbeckstraße 1, 55131 Mainz, Germany

E-Mail: [omar.hahad@unimedizin-mainz.de](mailto:omar.hahad@unimedizin-mainz.de)

Phone: +49 (0) 6131 17-2476

**Table S1.** Definition of covariates.

| Covariate | Definition |
| --- | --- |
| Socioeconomic status (SES) – score | Socioeconomic status was assessed by a validated index score (ranging from 3 to 21), providing information about educational background, current occupation, and salary (Lampert et al., 2013^1^). |
| Working night shift | Night shift work was defined as working hours between 11 p.m. and 5 a.m. |
| Time living at current residence in years | Participants were asked: “for how many years have you been living at your current residence?” |
| Current smoking | Participants’ self-reports were dichotomized into non-smokers (combining never smokers and former smokers) and current smokers (combining occasional and frequent smokers). Current smoking comprised regular or daily smoking (at least 1 cigarette per day, 7 per week, or 1 pack per month) for at least the past 6 months. The group of non-smokers included non-daily and non-regular smokers and former smokers who had a history of smoking (regular or daily smoking) for longer than 6 months and who were no current smokers. |
| Diabetes mellitus | Diabetes mellitus was defined by any of the following: diagnosis by a physician, antidiabetic treatment, fasting blood glucose level (overnight fast of at least 8 hours) ≥126 mg/dL, non-fasting blood glucose level (less than 8 hours of fasting) ≥200 mg/dL, or HbA1c ≥6.5%. |
| Hypertension | Arterial hypertension was diagnosed by the intake of antihypertensive drugs or a mean systolic blood pressure ≥140 mmHg or diastolic blood pressure ≥90 mmHg at rest (average of 2nd and 3rd standardized measurement after 8 and 11 minutes of rest). |
| Obesity | Obesity was defined as a body mass index ≥30 kg/m^2^. |
| Dyslipidemia | Dyslipidemia was present in the case of a physician diagnosis of dyslipidemia, low-density lipoprotein cholesterol/high-density lipoprotein cholesterol ratio >3.5, or triglycerides ≥150 mg/dL. |
| Family history of myocardial infarction or stroke | A positive history of myocardial infarction or stroke was recorded in a female first-degree relative ≤65 years or in a male first-degree relative ≤60 years. |
| Medication | Medication history was derived from medical records and personal reports and categorized according to the Anatomical Therapeutic Chemical Classification System (ATC) including intake of antihypertensives (C02), diuretics (C03), beta-blockers (C07), calcium channel blocker (C08), agents acting on the renin-angiotensin-aldosterone system (C09), and lipid modifying agents (C10). |
| ^1^Lampert T, Kroll LE, Muters S, Stolzenberg H. [measurement of the socioeconomic status within the german health update 2009 (geda)]. Bundesgesundheitsblatt Gesundheitsforschung Gesundheitsschutz. 2013;56:131-143. | |
